# Field Cage Assessment of Feeding Damage by *Riptortus pedestris* on Soybeans in China

**DOI:** 10.3390/insects12030255

**Published:** 2021-03-17

**Authors:** Wenjing Li, Yu Gao, Yinglu Hu, Juhong Chen, Jinping Zhang, Shusen Shi

**Affiliations:** 1 College of Plant Protection, Jilin Agricultural University, Changchun 130118, China; wenjingli321@163.com (W.L.); gaoy1101@163.com (Y.G.); hylhyl96220@163.com (Y.H.); 15181674153@163.com (J.C.); 2 MARA-CABI Joint Laboratory for Bio-safety, Institute of Plant Protection, Chinese Academy of Agricultural Sciences, No. 2 Yuanmingyuan West Road, Beijing 100193, China

**Keywords:** *Riptortus pedestris*, density, damage, soybean, development stage, quality

## Abstract

**Simple Summary:**

*Riptortus pedestris* (Fabricius) is a serious pest of soybeans. The nymphs and adults of *R. pedestris* cause feeding injury to soybean pods, resulting in significant field damage losses. In this paper, we tested the damage to soybeans at different growth stages caused by different densities of *R. pedestris*, through the field cage test. Our results showed that R_4_ was the most sensitive stage to *R. pedestris* injury. The damage intensity of the soybeans increased with increased pest density, and soybean nutrition factors changed after suffering *R. pedestris* injury. These results will be beneficial to future management programs of *R. pedestris* in soybean fields.

**Abstract:**

The bean bug, *Riptortus pedestris*, is a major pest of soybeans. In order to assess the critical stages of soybean damage by *R. pedestris*, we tested the damage to soybeans at different growth stages (R_2_, R_4_, and R_6_) caused by five densities of *R. pedestris* (1, 2, 3, 4, and 5) through a field cage experiment. The results show that the R_4_ stage was the most sensitive stage in terms of suffering *R. pedestris* injury damage, followed by the R_6_ stage and then the R_2_ stage. The number of stay green leaves was 7.04 per plant, the abortive pod rate of the soybeans was 56.36%, and the abortive seed rate of the soybeans was 46.69%. The dry weight of the soybeans was 14.20 g at the R_4_ stage; these values of R_4_ were significantly higher than at the R_2_ and R_6_ stages. However, the dry weight of soybean seed was 4.27 g and the nutrient transfer rate was 27.01% in the R_4_ stage; these values were significantly lower than in the R_2_ and R_6_ stages. The number of stay green leaves, abortive pod rates, and abortive seed rates were all increased significantly with increasing pest density at each stage of soybean growth. However, the nutrient transfer rate was significantly decreased with the increase in the pest density. Soybean nutrition factors changed after they suffered *R. pedestris* injury; the lipid content of the soybean seed decreased and the lipid content of the soybean plant increased compared to controls, when tested with a density of five *R. pedestris* in the R_4_ stage. These results will be beneficial to the future management of *R. pedestris* in soybean fields.

## 1. Introduction

The bean bug, *Riptortus pedestris* Fabricius (Hemiptera: Alydidae), is a piercing-sucking pest that causes serious damage to soybeans (*Glycine max*), as well as other economically important plants such as kidney beans (*Phaseolus vulgaris* L.), sesame (*Sesamum indicum* Linn.), rice *(Oryza sativa* L.), apples (*Malus domestica* Borkh.), and sweet persimmons (*Diospyros kaki* Thunberg) [1]. *R. pedestris* is widely distributed in South-East Asia, including in China, Korea, Japan, and India [2,3,4]. Two or three generations of *R. pedestris* occur per year depending on the climatic conditions of different areas [5,6], with three generations in North China and some parts of Korea [5,6,7]. Both the nymphs and adults cause damage to soybeans by inserting their needle-like mouthparts into the stems, leaves, flowers, pods, and seeds [8]. *R. pedestris* produce watery saliva that contains digestive enzymes during feeding, the same as the stink bug, by which the bean bug obtains nutrients and water [9]. The pods of soybeans drop or shrivel after suffering *R. pedestris* injury, and the transportation of nutrients is affected in damaged plants, resulting in “Staygreen Syndrome” [10,11]. Besides the above mentioned direct damage, *R. pedestris,* as a vector, disseminates yeast-spot disease [12], which often leads to the complete abortion of seeds in the whole field [13].

Soybeans are one of the four major oil crops in China. Soybean production is greatly affected by pentatomid feeding [14]. In the growth and development of soybeans, the vegetative stage involves the growth and development of the plant, and the reproductive stage is the development of soybean pods and seeds. The reproductive stage of soybeans was further defined into eight stages by Fehr and Caviness (1977) [15]. It has been reported that *R. pedestris* mainly injures soybeans during the reproductive stages [16,17,18].

Soybean seeds contain higher protein and lipid contents than other common legumes. They are the major source of protein in developing countries, as well as being important industrial raw materials [11]. Pod development is an important determinant for soybean yield, and pods play an important role in regulating the senescence process of soybean leaves [19]. Generally, hemipteran insect injury on seeds is carried out to complete their own development [20,21], causing the quality and yield loss of soybeans. *R. pedestris* injury leads to seed development stagnation; abortive seeds fail to produce signal substances to regulate leaf senescence and plant development, and hence the leaves cannot export photosynthates or receive senescence signals from the seeds, resulting in staygreen leaves and shriveled seeds [11]. In addition, soybean seeds change their chemical composition in response to infestation by stink bugs [22]. Soon et al. reported that the nutrient levels and seed germination of soybeans changed when *R. pedestris* feeding damage occurred in the R_5_ stage [23]. Here, we aimed to find the critical feeding damage stages of *R. pedestris* in soybeans by testing at the R_2_, R_4_, and R_6_ stages, as well as the density effect of *R. pedestris.* Furthermore, the nutritional changes in soybean plants and seeds in relation to an infestation of *R. pedestris* at the R_4_ stage was assessed.

## 2. Materials and Methods

### 2.1. Rearing of R. pedestris

The *R. pedestris* laboratory population was established from adults collected on soybean plants in Guiyang, China (26°30′15″ N, 106°39′19″ E). The colony was continuously reared in nylon mesh cages (45 × 45 × 45 cm) by providing soybean pods as food, refreshed every 3 or 4 days when needed. Newly emerged adults were regularly transferred to another cage. Colonies were maintained at 25 ± 1 °C, 65 ± 5% relative humidity, and a 16:8 h Light:Dark photoperiod.

### 2.2. Field Management of Soybean Plants

Two soybean plants were grown in one flowerpot (diameter = 28 cm; depth = 20 cm) containing normal soil, and covered with a nylon cage (22 × 12 cm), then set up on the ground outside the greenhouse at the teaching station of Jilin Agricultural University (43°48′47″ N, 125°25′4″ E). The plants were irrigated as needed to avoid water stress.

### 2.3. Releasing Different Densities of R. pedestris onto Three Development Stages of Soybeans

Three development stages of soybeans were tested: the full bloom stage (R_2_), full pod stage (R_4_), and full seed stage (R_6_). Males of *R. pedestris* at 2~7 days old were randomly selected from the rearing cages, and five insect densities (1, 2, 3, 4 and 5 per cage) were tested on the above soybean development stages. Three replications were carried out for each test.

### 2.4. Syndromes of Soybean Plants and Seeds Influenced by R. pedestris Feeding Damage

The aboveground parts of the soybean plants were cut using tree scissors at harvest season. The number of staygreen leaves, total pods, abortive pods, total seeds, and abortive seeds from each cage (including the control and treatment cages) was counted and recorded. Seeds were kept separately from each cage for weight, lipid, protein, and carbohydrate tests.

In staygreen leaves, the leaf color is green. An abortive pod is one in which a seed failed to fill the pod. An abortive seed was a seed that shriveled and became discolored.

The dry weight of soybean plants was taken by individually weighing the soybean plants, excluding the pods and roots, and they were dried in a drying oven at 80 °C. The soybean plants were weighed every eight 8 h by an electronic scale (PTX-FA300, Huazhi Scientific Instrument Co. Ltd., Fuzhou, China) until at a constant weight. The dry weight of the soybean seeds was measured in the same way.

Abortive pod rate (%) = Number of abortive pod/Total pods × 100

Abortive seed rate (%) = Number of abortive seeds/Total number of seeds × 100

The nutrient transfer rate (%) = Seed dry weight/(Seed dry weight + plant dry weight) × 100

### 2.5. Soybean Quality Influenced by R. pedestris Injury

The lipids of soybean seeds and plants were quantified by the soxhlet extractor method [24]. Soybean seeds and soybean plants were dried to a constant weight in a drying oven (ZXFD-15250 Jilin Jingke Instrument Equipment Co., Ltd. Changchun, China) at 80 °C for 3 d, and then kept in a desiccator for later testing. The dried soybean seeds or plants were ground to powder by a plant wall breaking machine (Y915s Jiuyang Co., Ltd. Jinan, China). Then, 2 g (W1) samples of the powder were added to the soxhlet extractor, and mixed with 90 mL petroleum ether to keep them at 80 °C in the water bath. We continued the extraction process for several hours, until the sample weight was constant. We waited for the samples to dry naturally, then weighed the sample again; weight (W2) was the sample without lipids. Lipid content (%) = (W1 − W2)/W1 × 100; W1 = 2 (g); W2 = the sample weight after extraction (g).

The protein of soybean seeds and plants was measured using a total protein quantitative test kit (Jiancheng Bioengineering Institute, Nanjing, China) follow the method of bicinchoninic acid (BCA) assay for protein quantitation [25], according to the instructions. Samples of 0.1 g of powder were mixed with nine times the volume of normal saline (0.1 mol/L, pH 7.4). Then, the mixed solution was mechanically homogenized in an ice water bath at 3500 R/min, centrifuging for 10 min. The supernatant was diluted into 1% tissue homogenate with saline solution. A total of 250 μL working reagent (test kit) was added into blank tubes, standard tubes, and sample tubes, respectively, and then they were kept at 37 °C for 30 min, before 750 μL termination solution was added into each tube for 5 min. The optical density (OD) value of each sample was determined by colorimetry at a 562 nm wavelength and 0.5 cm optical path. Total protein concentration (μg/mL) = (samples OD value—blank OD value)/(standard OD value—blank OD value) × standard concentration (563 μg/mL) × dilution ratio of the sample before testing.

The carbohydrates of soybean seeds and plants were quantified by anthrone methods [26]. Anthrone solution was prepared by dissolving 0.2 g anthrone in 100 mL of 80% sulfuric acid (the reagent was freshly prepared each day and used within 12 h). A total of 0.1 g dry powder was dissolved in 10 mL ddH_2_O and kept at 50 °C as a constant temperature in a water bath (W20m-2 Shellab, Hampton, USA) for 20 min, after which it was cooled and filtered. The filtrate was diluted to 250 mL, then 1000 μL ethanol was added and it was placed in the refrigerator at 4 °C overnight (about 16–17 h). We took the supernatant from the refrigerator and centrifuged it (Allegra X-22R (USA) for 20 min at 10,000 R/min. The supernatant was transferred into 20 mL test tubes, then 1000 μL 0.15 mol/L sulfuric acid solution was added, before the samples were transferred to a boiling water bath and hydrolyzed for 10 min. After cooling, we added 1000 μL 30% KOH solution and mixed evenly, before the samples were put in a boiling water bath for 10 min. Then, 1 mL test solution was added to 4 mL anthrone solution, before being transferred to a boiling water bath for 10 min then cooled by running water. After 20 min, colorimetry was performed at 620 nm with a spectrophotometer (SP-756P Shanghai Spectrum Instrument Co., Ltd., Shanghai, China) and the absorbance was recorded. The calculation formula of carbohydrate content: ①y = 0.0078x − 0.0068 ②A = (A0 × V × N)/(M × 1000); (y: absorbance value, x = A0: carbohydrate content from standard curve, A: carbohydrate content, V: volume of sample solution, N: dilution ratio, M: weight of sample).

### 2.6. Data Analysis

The impact of different plant stages and pest densities, as well as the interaction of these two factors, on staygreen leaves was analyzed using a generalized linear mode (GLM) with a Poisson distribution, followed by least significant difference (LSD) post hoc tests.

The impact of different plant stages and pest densities, as well as the interaction of these two factors, on the abortive pod rates, abortive seed rates, and nutrient transfer rates was also analyzed by GLM with a linear mode, followed by LSD post hoc tests.

The impact of different plant stages and pest densities, as well as the interaction of these two factors, on the dry weight of soybean plants and dry weight of soybean seeds was analyzed by two-way ANOVA. The dry weight of soybean plants and dry weight of soybean seeds at different soybean stages/different pest densities were analyzed by one-way ANOVA. Post hoc tests were followed by LSD.

The contents of lipids, protein, and carbohydrates were analyzed by independent samples t-test. All statistical analyses were carried out with the SPSS 21.0 ^®^ software package. All figures were produced by Origin 2018 software.

## 3. Results

### 3.1. Number of Staygreen Leaves

**Figure 1:**
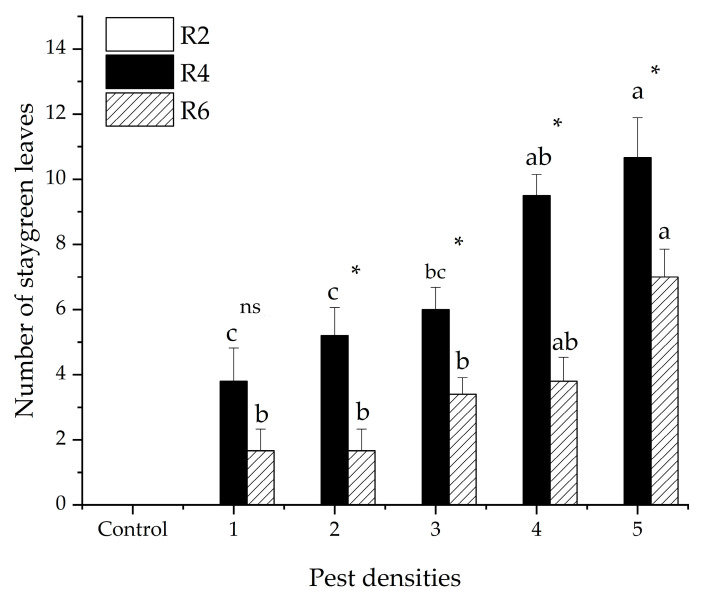
The number (mean ± SE) of staygreen leaves of soybeans caused by five pest densities during three soybean development stages (R_2_, R_4_ and R_6_). Pest densities are the row numbers of bean bugs. Note: The small letters denote significant differences among densities of pests in the same stage of soybean development, *p* < 0.05 (generalized linear mode (GLM)). * Means significant differences among different soybean development stages at the same density of pests at *p* < 0.05 (GLM). ns means no significant differences among different soybean development stages at same density of pests, *p* < 0.05 (GLM).

There were no staygreen leaves observed when *R. pedestris* fed at the R_2_ stage of soybeans at any tested pest density, or in the control. The number of staygreen leaves was significantly different among the R_4_ and R_6_ stages of soybeans (GLM *χ^2^* = 18.976, *df* = 1, *p* < 0.001). The average number of staygreen leaves was 7.04 ± 0.04 and 4.00 ± 2.51 for R_4_ and R_6_, respectively. The number of staygreen leaves significantly increased as the pest density increased (GLM *χ^2^* = 36.012, *df* = 4, *p* < 0.001). The average number of staygreen leaves was 3.00 ± 0.76, 3.86 ± 0.85, 4.82 ± 0.58, 6.33 ± 1.11 and 8.83 ± 0.90 when the plants were subjected to 1, 2, 3, 4, and 5 pests, respectively (Figure 1).

### 3.2. The Abortive Pod Rate

**Figure 2:**
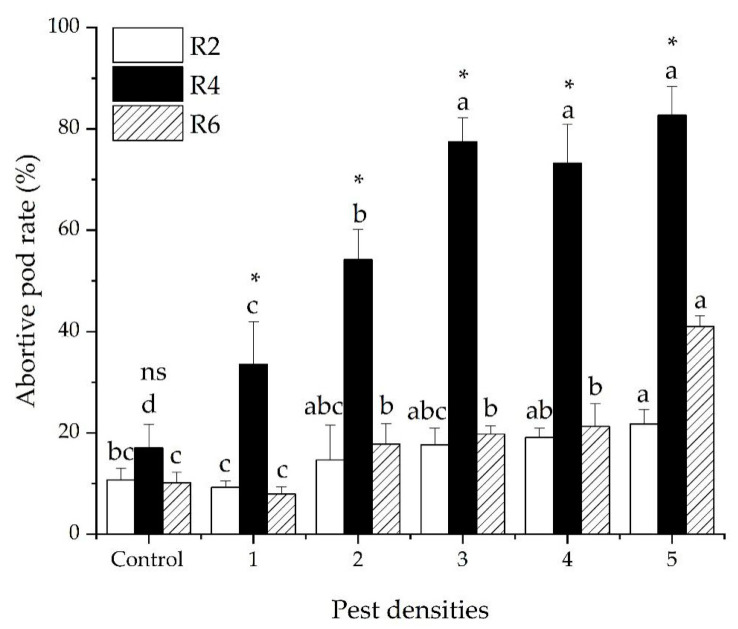
The percentage (mean ± SE) of soybean abortive pods caused by five densities of *R. pedestris* during three soybean development stages (R_2_, R_4_ and R_6_). Pest densities are the row numbers of bean bugs. Note: The small letters indicate significant differences among densities of pests in the same stage of soybean development, *p* < 0.05 (GLM). * Means significant differences among different soybean development stages at same density of pests, *p* < 0.05 (GLM). ns means no significant differences among different soybean development stages at same density of pests, *p* < 0.05 (GLM)

There was a significant interaction in the abortive pod rate of soybeans between pest density and the damaged stage of the soybeans (GLM *χ^2^* = 86.224, *df* = 10, *p* < 0.001). The ratios of abortive pods were significantly different among the three development stages of the soybeans (GLM *χ*^2^ = 106.602, *df* = 2, *p* < 0.001). The average ratios of abortive pods were 15.51 ± 1.55, 56.36 ± 4.74 and 19.67 ± 2.11% for R_2_, R_4_ and R_6_, respectively. The ratio of abortive pods significantly increased as the pest density increased (GLM *χ^2^* = 106.602, *df* = 2, *p* < 0.001). The average ratios of aborted pods were 16.92 ± 3.93, 28.89 ± 5.34, 38.29 ± 6.99, 37.87 ± 6.70, and 48.49 ± 6.52% when the plants were subjected to 1, 2, 3, 4, and 5 pests (Figure 2).

### 3.3. The Abortive Seed Rate

**Figure 3:**
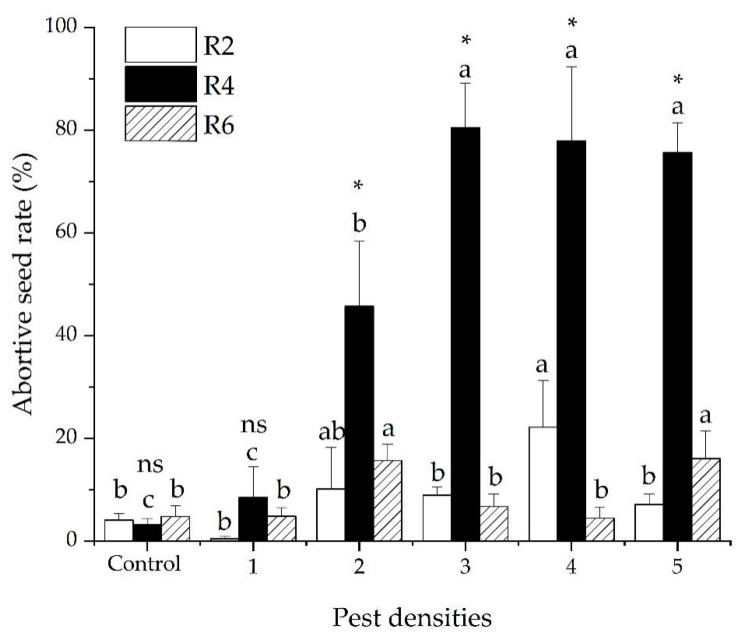
Soybean seed abortive rate (mean ± SE) following damage by five densities of *R. pedestris* during three soybean development stages (R_2_, R_4_ and R_6_). Pest densities are the row numbers of bean bugs. Note: The small letters indicate significant differences among densities of pests in the same stage of soybean development, *p* < 0.05 (GLM). * Means significant differences among different plant development stages at the same density of pests, *p* < 0.05 (GLM). ns means no significant differences among different plant development stages at the same density of pests, *p* < 0.05 (GLM)

There was a significant interaction in the abortive seed rate of soybeans between pest density and the damage stage of the soybean seeds (GLM *χ^2^* = 108.874, *df* = 10, *p* < 0.001). The ratio of abortive seeds was significantly different among the three soybean development stages (GLM *χ^2^* = 65.920, *df* = 2, *p* < 0.001). The average ratios of abortive seeds were 8.81 ± 0.02, 46.49 ± 2.91, and 8.76 ± 0.01% for R_2_, R_4_, and R_6_. The ratio of abortive seeds significantly increased as the pest density increased (GLM *χ^2^* = 24.124, *df* = 5, *p* < 0.001). The average ratios of abortive seeds were 4.59 ± 2.01, 23.83 ± 6.14, 32.04 ± 8.79, 34.86 ± 9.30, and 32.94 ± 5.75% when the plants were subjected to 1, 2, 3, 4, and 5 pests (Figure 3).

### 3.4. The Dry Weight

A significant interaction was found in the dry weight of soybean plants between pest density and the damage stages of soybeans (*F* = 5.060, *p* < 0.001). The dry weight of soybean plants was significantly different in the three plant development stages (*F*_(2, 105)_ = 17.720, *p* < 0.001). The average dry weights of soybean plants were 3.73 ± 0.14, 14.20 ± 2.08 and 7.46 ± 0.64 g for R_2_, R_4_ and R_6_, respectively. The dry weight of soybean plants significantly increased as the pest density increased (*F*_(5, 102)_ = 5.328, *p* < 0.001). The average dry weights of soybeans and leaves were 4.76 ± 0.80, 6.19 ± 0.67, 8.21 ± 1.42, 12.52 ± 3.24, and 14.78 ± 2.67 g when damaged by 1, 2, 3, 4, and 5 pests (Table 1).

There was a significant interaction in the dry weight of soybean seeds between pest density and the damage stages of soybeans (*F* = 2.120, *p* = 0.031). In addition, the dry weight of soybean seeds was significantly different in the three soybean development stages (*F*_(2, 105)_ = 26.830, *p* < 0.001). The average dry weights of soybean seeds per plant were 11.66 ± 0.57, 4.27 ± 0.81 and 9.40 ± 0.78 g for R_2_, R_4_ and R_6_, respectively. The dry weight of soybean seeds significantly increased as the pest density increased (*F*_(5, 102)_ = 6.178, *p* < 0.001). The average weights of soybean seeds were 10.09 ± 1.11 8.20 ± 1.46, 6.54 ± 1.06, 6.73 ± 1.15, and 5.83 ± 0.93 g when damaged by 1, 2, 3, 4, and 5 pests, respectively (Table 1).

### 3.5. The Nutrient Transfer Rate

**Figure 4:**
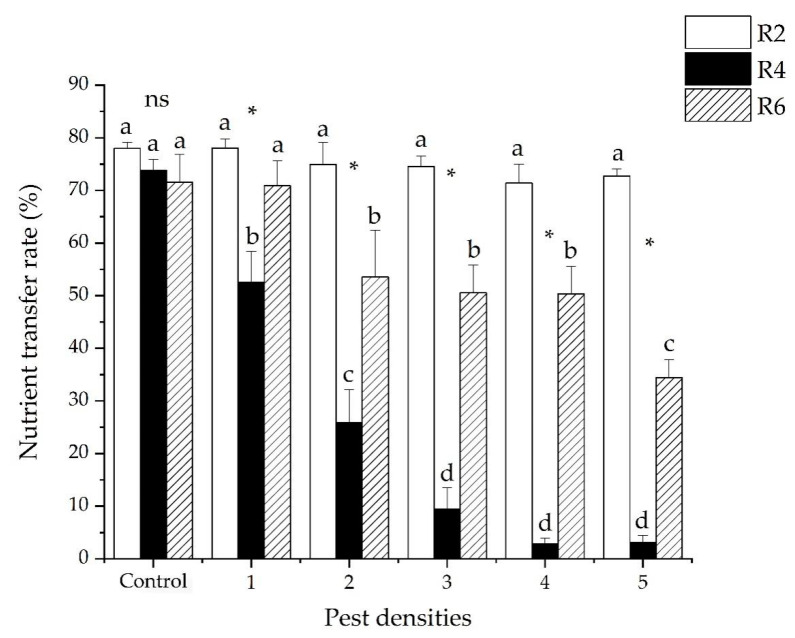
The nutrient transfer rate (mean ± SE) of soybeans when injured by five densities of *R. pedestris* during three soybean development stages (R_2_, R_4_ and R_6_). Pest densities are the row numbers of bean bugs. Note: The small letters indicate significant differences among densities of pests in the same stage of soybean development, *p* < 0.05 (GLM). * Means significant differences among different plant development stages at same density of pests at *p* < 0.05 (GLM). ns means no significant differences among different plant development stages at same density of pests at *p* < 0.05 (GLM).

There was a significant interaction in the nutrient transfer rate of soybeans between pest densities and the damage stages of soybeans (GLM *χ^2^* = 108.588, *df* = 10, *p* < 0.001). The ratio of nutrient transfer was significantly different in the three soybean development stages (GLM *χ^2^* = 114.632, *df* = 2, *p* < 0.001). The average ratios of nutrient transfer were 74.94 ± 1.08, 27.01 ± 4.54 and 55.22 ± 3.07% for R_2_, R_4_, and R_6_, respectively. The ratio of nutrient transfer significantly decreased as the pest density increased (GLM *χ^2^* = 32.757, *df* = 5, *p* < 0.001). The average ratios of nutrient transfer were 67.15 ± 3.57, 51.46 ± 6.09, 44.86 ± 6.88, 41.51 ± 7.25, and 41.52 ± 7.02% at 1, 2, 3, 4, and 5 pests, respectively (Figure 4).

### 3.6. Evaluation of the Nutrient Content of Soybean Plants and Seeds Caused by Five R. pedestris on the R_4_ Stage of Soybeans

The protein of soybean seeds showed no significant differences between the treatment and control (*t*_(1, 4)_ = 1.831, *p* = 0.234). The protein of soybean seeds was 0.16 ± 0.02 g/mL with five *R. pedestris,* and the control was 0.24 ± 0.05 g/mL. The protein of soybean plants was 0.23 ± 0.04 g/mL when tested with five *R. pedestris* at the R_4_ stage; it was significantly higher (*t*_(1, 4)_ = 5.587, *p* = 0.009) than the control (0.05 ± 0.00 g/mL). The lipids of soybean seeds were 0.11 ± 0.01 g/mL when tested with five *R. pedestris* at the R_4_ stage; this was significantly lower (*t*_(1, 4)_ = 3.212, *p* = 0.003) than the control (0.18 ± 0.00 g/mL). The lipids of the soybean plant were 0.04 ± 0.01 g/mL on the treatment; this was significantly higher (*t*_(1, 4)_ = 2.772, *p* = 0.009) than the control (0.01 ± 0.00 g/mL). The carbohydrate of soybean seeds was not significantly different between the treatment and control (*t*_(1, 4)_ = 0.386, *p* = 0.481), at 68.91 ± 21.47 g/mL and 94.87 ± 25.68 g/mL, respectively. The carbohydrate of soybeans was not significantly different between the treatment and control (*t*_(1, 4)_ = 0.754, *p* = 0.391), at 73.29 ± 7.77 g/mL and 81.73 ± 4.07 g/mL, respectively (Table 2).

## 4. Discussion

We found that the damage level of soybeans caused by *R. pedestris* was related to both the injury damage stage and the pest density. The number of staygreen leaves, abortive pod rate, abortive seed rate, and dry weight of the plants was increased with increasing pest density. In contrast, the rate of nutrient transfer and the dry weight of the seeds were reduced. The injury stage test results showed that R_4_ was the stage during which the most severe damage occurred to soybeans from *R. pedestris*, followed by R_6_, and then R_2_. Feeding injuries to soybeans caused by *R. pedestris* are similar to *Halyomorpha halys* (Hemiptera: Pentatomidae); the R_2_ stage is the least affected by *H. halys* feeding, while the R_4_ and R_6_ stage are similarly sensitive to injury [27]. In the R_4_ stage, related genes express and regulate soybean pod development, leaf development, physiological indexes, and hormone content [11]; this may be the mechanism by which plant leaves experienced staygreen in the R_4_ and R_6_ stages in our tests. The leaf is the source organ; it is the part that makes food and supplies nutrients to the other organs. *R. pedestris* feeding causes abortive pods and seeds of soybeans, and the severity increases with increasing pest density. The function of the leaves changes from photosynthetic source organs into sink organs. This leads to an imbalance of soybean sources and sinks, resulting in the blockage of the transportation of nutrients [28]. In addition, the seeds of the soybeans did not begin to develop in the R_2_ stage, which is the early stage of pod development, and the regulation of leaf senescence was not obvious in this stage [11]. Therefore, the R_4_ stage is the critical stage to control *R. pedestris* to avoid yield loss.

Protein, lipids, and carbohydrates are important components in crop growth and development. In our results, the protein content of the seeds was not significantly different between treatments and controls. However, the protein content of the plants treated with *R. pedestris* was significantly higher than the controls. Protein degradation is one of the basic characteristics of leaf senescence. The decrease in soluble protein content is caused by the increase in protease activity, which leads to the aggravation of protein hydrolysis [19]. Thus, we deduced that *R. pedestris* feeding resulted in staygreen syndrome due to the increased protein content in the plants. The lipid content in the treated soybean seeds was significantly lower than the controls; on the contrary, the lipid content of the soybean plants in the treatments was significantly higher than the controls. These results indicated that protein and lipid content changes in soybean pods/seeds infested by pentatomids result in qualitative and quantitative damage [9,29,30]. However, the lower lipid and carbohydrate contents of seeds cause a lower seed germination potential [23]. Therefore, *R. pedestris* damage to soybeans not only causes direct yield losses of soybeans, but also collateral loss of the next generation.

*R. pedestris* damage is mainly from piercing and sucking on pods, resulting in pod abortion as well as staygreen of stems and leaves of soybeans in harvest season [10]. The staygreen leaves, abortive pods, and abortive seeds of soybeans are the important parameters of staygreen syndrome. *R. pedestris* feeding on soybeans is a cause of staygreen syndrome, and leads to soybean yield decrease. Some control strategies have been suggested for *R. pedestris* to protect commercial crops. For instance, resistant strains can be selected for planting [31]. Sanitation of the field after harvest reduces the population of overwintering individuals [7]. Biological control is a sustainable and environmentally safe approach to management. *Gryon japonicum* (Hymenoptera: Scelionidae) and *Ooencyrtus nezarae* (Hymenoptera: Encyrtidae) are dominant egg parasitoids of *R. pedestris* [3,6]; they are promising bio-control agents against *R. pedestris*, although their release technology needs further research. Aggregation pheromone traps have a debatable impact on the monitoring and mass catching of *R. pedestris*. Endo et al. (2011) reported that populations of *R. pedestris* increased following trap attraction peaks in soybean fields [32]. However, Tabuchi et al. (2005) pointed out that pheromone traps are not an effective method of population monitoring [33]; on the contrary, damage to pods and seeds increased in plots with the placement of traps [34]. Chemical control has been shown to be an effective method [35]. Integrated pest management (IPM) strategies need to be explored for the sustainable management of *R. pedestris* based on the fact that R_4_ is the critical stage. We suggest the implementation of eco-friendly strategies to reduce the population of *R. pedestris* before stage R_4_ of soybeans.

## 5. Conclusions

We assessed the damage to soybean plants at different growth stages (R_2_, R_4_, and R_6_) caused by different densities of *R. pedestris* (1, 2, 3, 4, and 5) in field cage experiments. The results showed that R_4_ was the stage that suffered the most severe damage from *R. pedestris*. The positive parameters of soybean yield decreased as the pest density increased, and the negative parameters increased. Moreover, the nutrition of soybean seeds was decreased after *R. pedestris* injury. This research lays the foundation for the development of integrated pest management approaches for *R. pedestris* control in soybean fields, and we recommend that preventive measure be taken before the R_4_ stage.

## Figures and Tables

**Table 1:** Dry weight of soybean plants and seeds when damaged by different densities of *R. pedestris* during three soybean development stages.

Dry Weight	Development Stage of Soybean	Pest Densities					
		0	1	2	3	4	5
Soybean plants (g)	R_2_	3.76 ± 0.28 a A	3.24 ± 0.43 a A	3.97 ± 0.29 a B	3.60 ± 0.289 a B	4.26 ± 0.44 a C	3.56 ± 0.25 a C
	R_4_	4.66 ± 0.38 b A	6.41 ± 2.19 b A	7.72 ± 1.19 b A	14.02 ± 3.01 ab A	24.95 ± 7.44 a A	27.44 ± 3.82 a A
	R_6_	4.52 ± 0.64 b A	4.63 ± 0.75 b A	6.89 ± 1.23 b AB	7.01 ± 0.39 b B	8.34 ± 1.82 b B	13.33 ± 0.83 a B
Soybean seeds (g)	R_2_	13.84 ± 1.81 a A	11.89 ± 1.66 a A	13.26 ± 1.89 a A	10.63 ± 0.64 a A	10.83 ± 0.84 a A	9.48 ± 0.50 a A
	R_4_	13.29 ± 0.95 a A	6.31 ± 1.30 b A	2.60 ± 0.70 c B	1.31 ± 1.72 c B	1.12 ± 0.84 c B	0.96 ± 0.45 c C
	R_6_	12.66 ± 2.40 a A	12.08 ± 1.93 a A	8.74 ± 2.52 a AB	7.67 ± 1.24 a A	8.23 ± 1.45 a A	7.04 ± 0.85 a B

Note: The small letters denote significance among different densities of pests at the same stage of soybean development, *p* < 0.05 (ANOVA). Capital letters denote significant differences among soybean development stages with the same density of pests, *p* < 0.05 (ANOVA).

**Table 2:** Nutrient content of soybean plants and seeds at a density of five *R. pedestris* in the R_4_ stage.

Soybean	Treatment	Protein (g/mL)	Lipid (μg/mL)	Carbohydrate (μg/mL)
Seed	treatment	0.16 ± 0.02 a	0.11 ± 0.01 b	68.91 ± 21.47 a
	control	0.24 ± 0.05 a	0.18 ± 0.00 a	94.87 ± 25.68 a
Plant	treatment	0.23 ± 0.04 a	0.04 ± 0.01 a	73.29 ± 7.77 a
	control	0.05 ± 0.00 b	0.01 ± 0.00 b	81.73 ± 4.07 a

Note: The small letters denote significant differences between treatment and control of soybeans, *p* < 0.05 (independent samples *t*-test).

## Data Availability

The data presented in this study are available in this article results part.
